# The PLoS Community Journals

**DOI:** 10.1371/journal.pbio.0030129

**Published:** 2005-04-12

**Authors:** Mark Patterson, Catriona MacCallum, Hemai Parthasarathy, Caitlin Sedwick

## Abstract

2005 will see the launch of three new open access journals from the Public Library of Science - PLoS Computational Biology, PLoS Genetics, and PLoS Pathogens

The launch of *PLoS Biology*—only 18 months ago—was just a first step for the Public Library of Science. Our initial goal was to create a flagship journal for the broader PLoS mission by providing an open-access alternative to the best subscription journals in the life sciences, and to put open access firmly on the map.

Despite its youth, *PLoS Biology* is already becoming established as a publication of high standing, and *PLoS Medicine* is rapidly heading in the same direction. Submissions to *PLoS Biology* have steadily grown, recently surpassing the 1,000 mark, and every month sees international coverage of *PLoS Biology* articles in the media. With the increasing support for open access in general, the time is now ripe to build on the success of these two flagship journals by taking the next step and launching the PLoS community journals.

The PLoS community journals will give authors an opportunity to publish a greater range of high-quality papers in open-access journals, so that anyone can read, use, and build on their work. But these publications will also serve another function—they will provide examples that other journals can follow, and will increase confidence in the sustainability of open-access publishing as a business model. More publishers are exploring ways to remove existing subscription barriers and edge towards open access. And more funding agencies are expressing their commitment to make the findings of the research they support freely available to the public. The PLoS community journals will lend further weight to this inexorable shift towards open access.

Each PLoS community journal will cover a broad field of research—so the journals serve specific scientific communities. The journals are also run by the community—academic editors-in-chief and associate editors, supported by PLoS staff. And some of these journals will be collaborations with established community groups, such as the International Society for Computational Biology (ISCB), with whom we are partnering for the launch of *PLoS Computational Biology*. The twin strands of open-source software and public databases of biological information converge on this burgeoning discipline, and the case for an open-access journal in computational biology is easily made. The ISCB has taken a bold step, in the best interests of its discipline and membership. We hope that this action by the ISCB will inspire other scholarly societies to follow suit and will be only the first of many such collaborations between PLoS and other organizations.

The editorial teams running the first three PLoS community journals already comprise a group of over 80 researchers, each headed by an editor-in-chief: Philip E. Bourne (University of California, United States) for *PLoS Computational Biology* (www.ploscompbiol.org), Wayne N. Frankel (The Jackson Laboratory, United States) for *PLoS Genetics* (www.plosgenetics.org), and John A. T. Young (Salk Institute, United States) for *PLoS Pathogens* (www.plospathogens.org). The willingness of so many leading researchers to devote precious time to these new journals is testament to the level of commitment to open access that now exists within the research community, and the trust that PLoS has gained as a publisher of science and medicine.

And what of the relationship between the PLoS community journals and *PLoS Biology*? Does *PLoS Biology* still want papers in the areas in which PLoS launches new journals? Will *PLoS Biology* editors be rejecting more such papers in the knowledge that they will find a home in fellow PLoS publications? Let us assure you that we—the editors of *PLoS Biology*—remain committed to publishing the best research across all of biology.

Moreover, our relationship with the PLoS community journals is one of strict editorial independence. There is some overlap of membership on the editorial boards of *PLoS Biology* and the community journals, reflecting a level of dedication to open access, but as with all scientists who serve on multiple editorial boards and reviewers who review for multiple journals, these individuals are governed by confidentiality. That said, if an author would like a manuscript that has been turned down by one journal to be passed on to another, along with the reviewers' reports and their identities, we are happy to cooperate, subject to the permission of the reviewers. This can help to expedite the review process, saving time for authors, editors, and reviewers. The editors of *PLoS Biology* and all the PLoS journals are committed to offering a peer-review service that is as constructive, transparent, and efficient as possible.

In the world of scientific publishing, there is nothing quite like launching a journal, especially when the case for the journal is as strong as it is for the PLoS community journals. It's enthralling, nerve-racking, and relentless work. But when the manuscripts begin to arrive, the editorial process kicks into action, and the production team starts crafting the first accepted articles, it's hard to contain the excitement. PLoS is still a relatively small organization, and all our staff have played a part in preparing for the introduction of the new journals. But PLoS is much bigger than the people on the payroll. It's the research community that is making PLoS work, as demonstrated most emphatically by the editors-in-chief and editorial board members who have stepped up to launch the first three PLoS community journals. Please join us in making them a success, and enjoy your share of the excitement.

